# Detection of Rift Valley Fever Virus Interepidemic Activity in Some Hotspot Areas of Kenya by Sentinel Animal Surveillance, 2009–2012

**DOI:** 10.1155/2014/379010

**Published:** 2014-08-13

**Authors:** Jacqueline Kasiiti Lichoti, Absolomon Kihara, Abuu A. Oriko, Leonard Ateya Okutoyi, James Ogaa Wauna, David P. Tchouassi, Caroline C. Tigoi, Steve Kemp, Rosemary Sang, Rees Murithi Mbabu

**Affiliations:** ^1^Ministry of Agriculture Livestock and Fisheries, P.O. Box 00625, Nairobi, Kenya; ^2^International Livestock Research Institute, P.O. Box 30709–00100, Old Naivasha Road, Nairobi, Kenya; ^3^Kenya Agricultural Research Institute, Biotechnology Centre, P.O. Box 57811-00200, Waiyaki Way, Nairobi, Kenya; ^4^International Centre of Insect Physiology and Ecology, Human Health Division, P.O. Box 30772-00100, Nairobi, Kenya; ^5^Kenya Medical Research Institute, Centre for Virus Research, P.O. Box 54628-00200, Nairobi, Kenya

## Abstract

Rift Valley fever virus causes an important zoonotic disease of humans and small ruminants in Eastern Africa and is spread primarily by a mosquito vector. In this region, it occurs as epizootics that typically occur at 5–15-year intervals associated with unusual rainfall events. It has hitherto been known that the virus is maintained between outbreaks in dormant eggs of the mosquito vector and this has formed the basis of understanding of the epidemiology and control strategies of the disease. We show here that seroconversion and sporadic acute disease do occur during the interepidemic periods (IEPs) in the absence of reported cases in livestock or humans. The finding indicates that previously undetected low-level virus transmission during the IEPs does occur and that epizootics may also be due to periodic expansion of mosquito vectors in the presence of both circulating virus and naïve animals.

## 1. Introduction

Rift Valley fever (RVF) is an acute mosquito-borne viral zoonosis affecting ruminants and humans [1]. It occurs in the form of epizootics/outbreaks that come in cycles of 5 to 15 years following heavy persistent rainfall with flooding that result in abundance of flood water mosquitoes known to transmit the virus [2]. The RVF virus belongs to the genus* Phlebovirus* of the family Bunyaviridae and causes high fever and abortion in susceptible pregnant animals irrespective of the gestation period and high mortality in newborns. The disease manifests in humans as asymptomatic or mild illness with headache, fever, and muscle and joint pains to severe illness associated with hemorrhagic fever, encephalitis, or ocular disease [3, 4]. In addition to infected mosquito bite [5–8], humans may be infected through handling of blood and tissues of viremic livestock. Rift Valley fever is associated with unpredictable human epidemics in Africa [9] and often involves several countries in the East African region at the same time [10].

During outbreaks, the disease seriously affects rural people in terms of food security and household nutrition through direct and indirect losses to livestock production. The psychosocial distress that communities go through is enormous, resulting from loss of family members and/or relatives to disease, and general livelihoods due to ban on livestock movement and livestock product trade and/or ban on export affecting the economic status of most livestock producers [11].

RVF maintenance between epidemics is not fully understood largely because detection of the virus during the interepidemic period (IEP) has proven difficult. It has been widely hypothesized that it is maintained through transovarial transmission in the floodwater mosquitoes of the* Aedes* genus [12–14]. The unpredictable nature of outbreaks highlights the need for active surveillance to monitor circulation of the virus especially during the IEPs [4] to obtain an improved understanding of the mechanisms of disease transmission to ruminants and subsequent spillover to humans which leads to outbreaks. There have been reports of interepidemic circulation of RVFV in endemic countries in Africa [15] in a number of Eastern Africa countries. Sporadic cases of RVF that occur in such endemic zones with manifestation of abortions could easily be confused for other causes of abortion in livestock and be missed out [16]. Circulation of RVFV in the absence of clinical disease among livestock has also been reported in some of the countries in the region.

Virus circulation occurring among apparently healthy animals has also been observed in Somalia [17], Uganda [18], Mayotte [13], Tanzania [15], and Madagascar [19] and also in wild animals [20]. RVF antibodies have also been detected in Kenya in areas where the disease has never been reported [10] further confirming virus circulation without clinical disease. Using modeling, it has been shown that RVF virus can persist in an environment as long as the virus remains in a mosquito breeding site once introduced [21].

Ruminants such as goats and sheep have been used as sentinels for surveillance of RVF virus activity [12, 22, 23] involving the detection of antibodies against the virus in sera. In Kenya, this approach has been mainly limited to suspected sick or aborting animals [24] and the extent to which asymptomatic but viremic animals may act as reservoirs of infection is unknown. Monitoring of infection in susceptible hosts is therefore an important tool for risk assessment. Furthermore, data on species susceptibility and on animal demographics can provide useful information in identifying RVF risk areas and variation [25].

This study reports on the monitoring of RVF virus activity in sentinel herds is comprised of sheep and goats. This work was conducted as part of a project geared towards understanding the interepidemic circulation of the virus in diverse host systems and focusing on sites that were recently affected by the RVF outbreaks in 2006/2007. We sought to determine the degree and variation of RVF virus exposure in these herds including variation across the selected periods and sites.

## 2. Materials and Methods

### 2.1. Study Area

**Figure 1:**
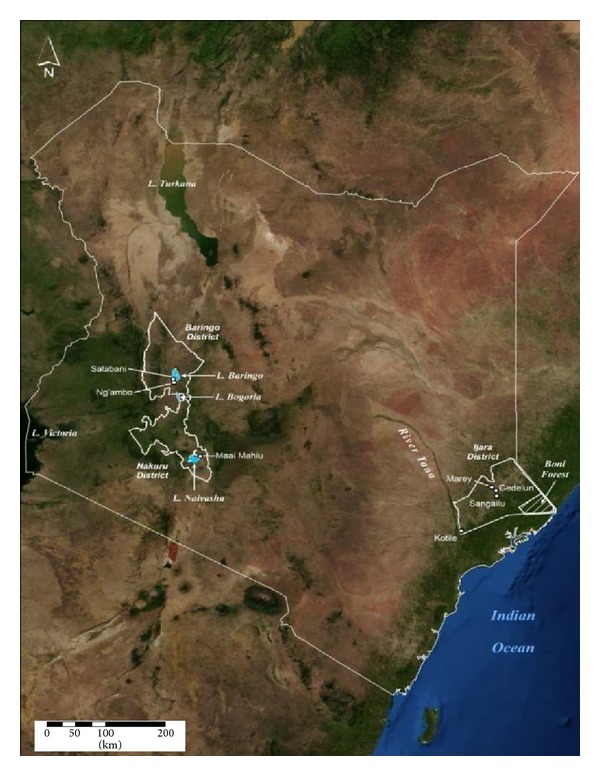


This study was conducted between 2009 and 2012 in Naivasha, Ijara, and Baringo Districts of Kenya. These sites were selected due to historic occurrence of epizootics/epidemics of RVF in 1997/1998 and 2006/2007 except for Naivasha where the disease is endemic. Ijara and Baringo districts are both semiarid with low erratic rainfall but prone to flooding in times of heavy persistent rainfall. They are home to predominantly pastoralist communities which maintain large livestock herds. However, the regions are geographically far apart in the North Eastern part of Kenya lying close to the Kenya-Somalia border to the North East and Boni Forest and the Tana Delta to the South while Baringo is in the Central Rift Valley of Kenya lying at the shores of Lake Baringo. Whereas RVF epizootics have repeatedly occurred in Ijara, an outbreak of RVF was reported for the first time in Baring in 2006/07. Through the review of records at the district and guidance from the district veterinary officer (DVO) and the medical officers of health, locations which reported cases of RVF during the outbreaks in humans and animals were identified for the study. Sentinel herds that had no history of RVF vaccination were set up in the selected locations in the districts as indicated in Figure 1. Three sites were identified to represent Ijara, two towards the North of Boni forest area (Ijara (Sangailu/Gedelun centered on 1.3148°S, 40.7327°E/1.3837°S, 40.7133°E)) and one towards the shores of the Tana Delta (Kotile 1.9658°S, 40.2063°E), two in Baringo (Ng'ambo: 0.4940°N, 36.0588°E and Salabani: 0.5507°N, 36.0501°E) around Lake Baringo and one in Naivasha (0.7203°S, 36.4284°E) at the base of the escarpment (Figure 1).

In villages, at livestock/wildlife interface and in close proximity to forested areas around the lake Baringo where pasture is available during dry season, with livestock grazing in proximity to wild animals and returning to villages during the rains. The herds identified had over 300 animals and there was frequent contact between livestock and wildlife according to information given by the local community. Using the government and community leaders, the herd owners were given the background and objectives of the study. Their role in the project was to provide a subset of their herd/animals to be tagged for sentinel surveillance. The selected animals in the herd were to continue to move and graze together with the rest of the animals.

Farmers who agreed to take part in the study by allowing their animals to be tagged and sampled signed a consent form and a letter of agreement with the project in the presence of a witness, the local administration, local veterinary officer, and the livestock owner's representative. This stipulated in detail how the sentinel herd would be managed by both the livestock owners and the project. Funds were set aside for purchasing of antihelminthics for all the animals in the herds, catering for all the veterinary services, and payment of herding fee for the recruited animals during the project period.

### 2.2. Selection of Sentinel Animals

Young sheep and/or goats aged 12 to 15 months were identified and permanently marked using ear tags. Ageing of the animals was based on the presence of the incisor permanent teeth. Young females were preferred because they are kept longer for increasing herd size through lambing and kidding thus are rarely sold. After tagging, the baseline blood samples were collected. All samples were tested for antibodies (IgG & IgM) to RVFV for baseline data. All selected tagged animals were included in the study irrespective of whether positive or negative for RVF antibodies. During each visit, the missing animals were replaced guided by the selection criteria.

The number of animals selected at each site for each sentinel herd was maintained at above the recommended 40 RVF seronegative animals (Table 1) per location. This was attained by recruiting new additional animals during each visit to replace those that died or got sold or seroconverted.

### 2.3. Frequency of Sample Collection

After the baseline sampling, animals were sampled every 4–6 weeks up to the end of the rainy season during short and long rains. The number of visits depended on how long the rainy season persisted. Samples were forwarded to Central Veterinary Laboratory, Kabete, Nairobi, to be tested for the presence of RVF virus-specific IgG and IgM antibodies.

Besides sentinel serological surveillance, other animals in the neighbourhood were monitored for any clinical disease suggestive of RVF. The case definition for RVF infection was animals showing abortions, mortality in lambs and kids, high fever, lymphadenitis, nasal and ocular discharges, profuse fetid diarrhoea, severe prostration, dysgalactia, and jaundice. In instances where some of these clinical signs were encountered, EDTA blood (10 mL) was collected and transported frozen to the laboratory for analysis.

### 2.4. Sample Collection, Transportation, and Processing

Blood was collected aseptically from the jugular vein into 10 mL EDTA vacutainers and into plain vacutainers precoated with serum activator. The animal unique identification (id) on ear tag was recorded to link the vacutainer code with the animal identification. The vacutainers for whole blood were placed in a cool box with dry ice and transported to the laboratory for aliquoting. Later on, they were aliquoted into 3 bar-coded cryotubes, and samples were placed on dry ice ready for transportation. The blood for serum was slanted in the shade to prevent exposure to excessive temperatures. These were then taken to the refrigerator and placed vertically until the following day. The tubes were carefully removed from the refrigerator and centrifuged, the bungs were removed, and clear serum was carefully pipetted off into sterile bar-coded cryovials. The sample identification was then entered and linked to the animal identification for accurate data maintenance.

### 2.5. Animal Data Capture and Storage

A 10-inch Hewlett Packard (HP) netbook, a USB Bluetooth adapter (UD100), a USB GPS Dongle (ND100S), a 1-dimensional barcode scanner, radio frequency identification (RFID) tags, and a hand held RFID tag reader were used for animal data capture and storage in the field.

Sentinel animals were ear tagged with radio frequency (RFID) chips (All flex, USA). A Bluetooth enabled RFID reader was used to capture the animal identity during sampling and to transmit this information via Bluetooth to a netbook.

In order to ensure error-free sample collection, a software system (tarakibu) was developed using python, html and JavaScript, and MySQL to associate the animal id, GPS-derived time and location at sampling time, and to associate sample barcodes into a database. The aliquoting process was managed by a program (Ukasimu) which ensured that barcodes on aliquots were associated with the original collection data. Further information on these systems including source code is available at https://github.com/ilri/tarakibu and https://github.com/ilri/ukasimu.

### 2.6. Laboratory Analysis of Samples

The serum samples were analyzed using the inhibition enzyme-linked immunoassay kit for the detection of antibody (both IgG and IgM) to Rift Valley fever virus in humans and domestic and wild ruminants (Special Pathogens Unit, National Institute for Communicable Diseases, South Africa) [26]. The positive serum samples for RVFV virus-specific antibodies were then tested for IgM antibodies using the IgM capture enzyme-linked immunoassay (BDSL, Special Pathogens Unit, National Institute for communicable Diseases, Johannesburg, South Africa) [27]. The kits were used following the protocol of the manufacturer. Whole blood from animals that tested positive on IgM capture ELISA was tested for RVFV antigen by quantitative real time RT-PCR (qRT-PCR) using RVF reagents from CDC Atlanta [28] and the Applied Biosystems 7500 real time PCR equipment was used. The primer and probe set used was RVFL-2912fwdGG (5′-TGAAAATTCCTGAGACACATGG-3′), RVFL-2981revAC (5′-ACTTCCTTGCATCATCTGATG-3′), and RVFL-probe-2950 (5′-CAATGTAAGGGGCCTGTGTGGACTTGTG-3′) labeled at the 5′ end with the reporter dye FAM and at the 3′ end with the quencher BHQ1. The target region was the L segment. The samples that tested positive on RVF Inhibition ELISA but tested negative on RVF IgM capture ELISA were presumed to be positive for IgG antibodies. The purpose was to save on laboratory testing costs and only pick RVF IgM positives that indicated current exposure of the animals to RVF virus.

### 2.7. Data Analysis

The number of seropositive animals out of the total sampled for each period was analysed using a generalized linear model (GLM) with binomial error structure or quasibinomial error structure in case of overdispersion and log link in R 2.11.0 software [29]. When any of the parameters (animal type, site, or sampling period) was significant, the odds ratio (OR) and corresponding confidence interval (CI) were estimated against a reference category from each type.

## 3. Results

### 3.1. RVFV Seroconversion in Ijara

In Kotile, the percent seropositivity of the animals oscillated between 0–2.5% during the period of study. On every visit, animal replacements were more frequent because, at this site, animals were recruited from 5 different livestock owners and challenges to present the animals for sampling by some owners resulted in missed sampling opportunities and the need for replacement (Table 2). In Sangailu, animals were recruited from one livestock owner and replacement was mostly due to seroconversion.

There was a slight decrease in seropositivity (from 5.3% to 3.6%) between September and October 2009, but when the animals were sampled in early February 2010, 39 out of 56 (69.6%) animals had antibodies against RVFV with only 17 testing negative. Only one animal was replaced during this period. Samples collected during targeted surveillance in October 2010 from 2 goats that showed clinical signs of RVF infection at a homestead in Marey (Ijara) were positive for IgM antibodies by ELISA and also antigen positive on real time qRT-PCR. The owner of the Sangailu herd had removed all the ear tags from the animals on the subsequent visit; as such, more animals were retagged and samples were collected for testing and eight were positive for RVFV antibodies during the sampling of May 2010. The next sampling carried out at the end of June and early July 2010 showed a seroconversion of 48.2%. This necessitated the recruitment of another herd (Gedulun herd) in the same area in July 2010 of which 7 of the 50 animals had seroconverted when the second sampling was carried out in October 2010 (Table 2).

Analyses of the combined data from Ijara showed that there was no significant difference in seropositivity between the animal types (goat and sheep) (*F*_1,14_ = 2.089, *P* = 0.170) and sampling period (*F*_3,12_ = 0.7806, *P* = 0.527). However, there was a significant difference in the level of virus exposure between the two sites (*F*_1,14_ = 13.702, *P* = 0.002) with about 35-fold greater likelihood of exposure recorded in Sangailu relative to Kotile (95% OR = 33.71, CI = 18.096–76.686). Analyses of combined available data for Marigat district during the sampling periods May 2009, November 2009, January 2010, April 2010, May 2010, April 2011, August 2011, and October 2011 showed no significant difference in exposure between the animal types, goat and sheep (*F*_1,8_ = 2.766, *P* = 0.130); sites, Ng'ambo and Salabani (*F*_1,8_ = 2.766, *P* = 0.135); and sampling period (*F*_4,5_ = 2.619, *P* = 0.160).

We also analysed risk of RVF exposure for each site individually. Our findings showed an overwhelmingly significant difference in RVF exposure between the animal types (*χ*2_1,8_ = 9.322, *P* < 0.001) in Kotile with about a 51-fold risk of infection recorded in sheep compared to goat (OR = 51.51, CI = 13.507–337.001). However, there was no significant difference recorded across the sampling periods (*F*_6,3_ = 0.422, *P* = 0.830). A converse pattern was observed in Sangailu where there was no significant difference in exposure between the animal types (*F*_1,8_ = 0.109, *P* = 0.749) and sampling period (*F*_4,5_ = 4.6366, *P* = 0.0617), although, with respect to sampling periods, risk of RVF exposure was highest in February-2010 in Sangailu as seen in Table 2.

### 3.2. RVFV Seroconversion in Naivasha

In Naivasha, 106 animals were recruited to the study (Table 1) and, during the baseline sampling, only five animals had antibodies to RVF virus. The subsequent samplings had zero, one, four, two, and one animals testing positive for RVF antibodies. There were always replacements during each visit (Table 3).

There was no significant difference in exposure between the animal types (*F*_1,10_ = 0.9096, *P* = 0.363) although there was about a 2-fold higher likelihood of infection to occur in sheep when compared to goat (95% OR = 1.81, CI = 0.584–6.749). Also, the risk of exposure did not seem to differ across the sampling period (*F*_5,6_ = 3.020, *P* = 0.106).

### 3.3. RVFV Seroconversion in Marigat

In Ng'ambo, the livestock owner had two types of herds: goats that graze near the homestead in the Perkerra irrigation scheme and sheep that left to graze around the shares of Lake Baringo. During the short rains period of November 2009, 26 (26.5%) were positive in Ng'ambo and 8 (10.5%) were positive in Salabani. The next sampling carried out after six weeks revealed 24 (24.4%) positives in Ng'ambo and 11 (14.1%) positives in Salabani. During the sampling in January 2010, some of the animals in Ng'ambo were not presented for sampling because they could not be traced in the expansive thickets of* Prosopis juliflora* at the time.

There was no significant difference in exposure due to animal type both in Ng'ambo (*F*_1,12_ = 2.4491, *P* = 0.1176) and Salabani (*F*_1,14_ = 0.465, *P* = 0.50). Interestingly, the risk of infection significantly differed across the sampling periods at both sites. The highest risk of exposure was recorded during the periods of November 2009 and January 2010 at both sites relative to the others (Table 4).

### 3.4. Active RVFV Infection

All the positive samples on RVF inhibition ELISA were tested using the IgM capture ELISA. Real time qRT-PCR was carried out on the samples that were positive on RVF IgM capture. One sheep from the sentinel herd in Kotile was positive for RVF IgM antibodies and RVF antigen by qRT-PCR. Two samples from opportunistic sampling were positive, one from Ijara and the other from Marigat, both by IgM ELISA and qRT-PCR. All the other positive samples on RVF Inhibition ELISA were negative for IgM antibodies (Table 5).

## 4. Discussion

This study detected evidence of RVFV circulation during the IEP 2009 to 2012 in Ijara and Marigat (sites where RVF outbreaks affecting livestock and humans were reported in 2006/07) [30] with acute virus infections being detected among sentinel animals and among animals sampled opportunistically within the vicinity. There were no reports of human RVF and no reports of increased abortion or suspected RVF outbreak by the veterinary and public health authorities in these areas. Interepidemic transmission of RVFV was also demonstrated through seroconversion among the herds also in the absence of reports of clinical cases or reports of outbreaks among local animals and human populations. The observation of RVFV circulation among livestock in the IEP in Kenya is based on both detection of infection and seroconversion which would have escaped the knowledge of the veterinary and public health authorities raising concerns about capacity for alertness and preparedness in these hotspots and the need for improved detection of RVFV activity among livestock.

Animals from Ijara (mainly cattle) and Marigat (even sheep) move to the forest in Boni and around Lake Baringo, respectively, when pasture and water become scarce, returning back to the normal grazing areas/villages when rains return and pasture becomes available. In both study sites, the increase in the seroprevalence was associated with flooding in the areas and animals migration back from the forest after dry spells. The forest ecosystem may be playing a role in the interepizootic maintenance or amplification cycle for RVF in Kenya as postulated by Davies [2] who observed a small percentage of seroconversion in cattle that grazed in forested areas and the role played by wildlife [20].

The observed RVFV activity in Baringo in this IEP (2009 to 20012) suggests that the virus may have been established in the area earlier even though outbreaks were reported here for the first time during the 2006/07 Kenya outbreak. This gives credence to the argument that the virus may not have been introduced in the area in 2006/07 through movement of infected livestock from Northeastern Kenya during the outbreak but rather occurred* de novo* in Baringo [10]. This means that the virus may have been here earlier than we know.

Minimal virus circulation was detected in Naivasha herd over the monitoring period. Naivasha was selected to represent a site classified as endemic which experiences low level virus activity every rainy season and not explosive outbreaks such as those reported in Ijara and Marigat in 1997/98 and 2006/07. The herd was hosted by a farmer whose animals are confined and do not migrate to forests in search of pasture and, in addition, the cattle were reported to receive RVF vaccination during periods of high risk which reduced the risk of exposure of our sentinel herds.

The observed significant difference in risk of exposure/infections in sheep compared to goat in Kotile as opposed to Sangailu raises questions on differential risk levels attributable to differences in animal species/breeds. It has often been suggested that sheep are more susceptible to RVF than goats with more sheep being affected than other animals during outbreaks [31–34]. However, this indicates differences in exposure risk between the sheep populations from Kotile and Sangailu and further suggests difference in virus exposure risk between sheep breeds or populations.

Following up the sentinel herds from 2009 to 2012, it was observed that the level of replacement was high in all the study sites owing to loss of recruited animals through death, disappearance and seroconversion. This greatly compromised following up of individual animals making it a costly venture when, at every visit, animals had to be replaced and tagged. In light of this challenge, we recommend that sentinel animals be followed up as a herd rather than at individual level and criteria for selecting animals be sampled developed. This could include sampling specific age of animals whose history of vaccination against RVF is known. Following up a herd rather than an individual animal would be more sustainable and cost effective compared to longitudinal following up of individual animals. There was also the challenge of livestock owners thinking that they would be victimized when their animals were tagged. This caused some of them to remove the ear tags as the case was in Sangailu which contributed to interruptions in the follow-up.

Over the entire sampling period, risk of exposure was significantly higher based on the sampling done in October 2009 and January 2010 for Marigat (Ng'ambo and Salabani) and in February 2010 for Sangailu which may be attributed to the amounts of rainfall and flooding that occurred during these periods usually associated with short rains period in the country. It is worthy of note that most outbreaks have been associated with short rains which occur in above normal amounts [10, 30, 35–37].

From the observations made from following the herds in Ijara (Sangailu, Gedulum) and Marigat (Salabani and Ng'ambo), it can be concluded that RVF virus activity occurs even in the absence of clinical infections in herds [35, 37–39]. Active surveillance is needed between epidemics to be able to detect transmission among livestock and possible human exposure that may go undetected among remote rural communities. Most veterinary authorities depend on passive surveillance to detect diseases that do not commonly occur. This is widely accepted because it has served the purpose thus far. Passive surveillance is not easy to detect in the current case of RVF virus circulation when animals are not showing clinical signs. Active surveillance is recommended and, where resources may not be available, targeted surveillance in high risk areas will help curb RVF outbreaks. Isolation of virus causing outbreaks is important in order to detect whether there are any variations [39, 40]. There is also a need to relook the contingency plan used in response to RVF outbreaks, bearing in mind that active transmission of the virus could occur in the absence of expected clinical events that have been relied on for a long time such as massive abortions in livestock.

## Figures and Tables

**Table 1:** Ijara to be at same level with Sangailu because it represents Sangailu, Gedelun and Kotile as shown below.

District	Site	Sheep	Goats	Total
Naivasha	Mai Mahiu	59	47	106
Ijara	Sangailu	25	31	56
	Gedelun	50	—	50
	Kotile	17	139	156
Marigat	Ng'ambo	50	48	98
	Salabani	53	25	78
Total		**254**	**290**	**544**

**Table 2:** Number of RVF seropositive animals and number of replaced animals during the different sampling periods at Ijara district.

Site	Animal type	Sampling period						
		September 2009	October 2009	February 2010	May 10	June-July 2010	October-November 2010	June 2012
Kotile	Sheep (*n*)		2 (17)	3 (17)	0 (17)	3 (17)		3 (17)
	Goat (*n*)		1 (139)	0 (139)	0 (139)	0 (139)		1 (139)
	Number replaced			36	12	49	13	

Sangailu	Sheep (*n*)	2 (25)	2 (25)	25 (25)	2 (25)	9 (25)		
	Goat (*n*)	1 (31)	0 (31)	14 (31)	6 (31)	18 (31)		
	Number replaced			1	56			

Gedelun	Sheep (*n*)					0 (50)	7 (50)	1 (50)
	Goat (*n*)					—	—	—
	Number replaced					4	14	

Numbers in brackets (*n*) indicate total number sampled for each animal per sampling period; — shows data on animals not recruited.

**Table 3:** Number of RVF seropositive animals and number of replaced animals during the different sampling periods in Naivasha.

Animal type	Sampling period					
	January 2010	May 2010	June 2010	January 2011	July 2011	December 2011
Sheep (*n*)	4 (59)	0 (59)	1 (59)	3 (59)	1 (59)	0 (59)
Goat (*n*)	1 (47)	0 (47)	0 (47)	1 (47)	1 (47)	1 (47)
Number replaced	18	34	39	13	14	0

Numbers in brackets indicate total number (*n*) sampled for each animal per sampling period.

**Table 4:** Number of RVF seropositive animals and number of replaced animals during the different sampling periods at Marigat district.

Site	Animal type	Sampling period									
		May 2009	November 2009	January 2010	April 2010	May 2010	April 2011	August 2011	October 2011	March 2012	May 2012
Ng'ambo	Sheep (*n*)		11 (50)	10 (50)	1 (60)			1 (49)	12 (50)	5 (48)	3 (54)
	Goat (*n*)		15 (48)	14 (48)	2 (55)			1 (49)	10 (46)	5 (52)	8 (46)
	Number replaced			8	21	42	22	24	—	—	—

Salabani	Sheep (*n*)	1 (51)	4 (53)	7 (53)	0 (53)	0 (53)	3 (53)	0 (53)	1 (51)		
	Goat (*n*)	0 (25)	4 (25)	4 (25)	1 (25)	0 (25)	1 (25)	0 (25)	0 (25)		
	Number replaced			9	17	15	14	27	—	—	—

Numbers in brackets indicate total number (*n*) sampled for each animal per sampling period.

**Table 5:** Animals with evidence of recent RVF infection (sentinel and targeted surveillance).

Animal ID	Species	Sample	Location	Sampling date	Type	IgM ELISA	qRT-PCR
AVD1002	Sheep	Serum	Marey Sangailu	28.10.09	Targeted surveillance	Positive	
AVD1002	Sheep	Whole blood	Marey/Sangailu	28.10.09	Targeted surveillance		Positive
AVD063	Sheep	Serum	Kotile	29.10.09	Sentinel	Positive	
AVD063	Sheep	Whole blood	Kotile	29.10.09	Sentinel		Positive
AVD392	Goat	Serum	Salabani	5.11.09	Targeted surveillance	Positive	
AVD392	Goat	Whole blood	Salabani	5.11.09	Targeted surveillance		Positive
